# Intent to Test for COVID-19 in the Postpandemic Era

**DOI:** 10.1001/jamanetworkopen.2025.18250

**Published:** 2025-06-30

**Authors:** Kimberly A. Fisher, Kathleen M. Mazor, Mary T. Antonelli, Caitlin Pretz, Yanhua Zhou, Apurv Soni

**Affiliations:** 1 UMass Chan Medical School Department of Medicine, Division of Health Systems Science, Worcester, Massachusetts; 2 UMass Chan Medical School Department of Medicine, Division of Pulmonary and Critical Care Medicine, Worcester, Massachusetts; 3 UMass Chan Medical School Department of Medicine, Program in Digital Medicine, Worcester, Massachusetts; 4 Tan Chingfen Graduate School of Nursing, Division of Health Systems Science, UMass Chan Medical School, Worcester, Massachusetts

## Abstract

This cross-sectional study used an online national survey to examine the intent to test when COVID-19 was suspected among adults in the US between October 2024 and April 2025.

## Introduction

COVID-19 is a continuing health threat, with the Centers for Disease Control and Prevention estimating 28 000 to 46 000 deaths and 230 000 to 390 000 hospitalizations due to COVID-19 in the US between October 2024 and April 2025.^1^ Early identification of infection enables prompt care and steps to reduce spread. Timely initiation of oral antiviral medications is associated with lower hospitalizations, deaths, and long-COVID incidence among adults at high risk.^2^ Although motivation to self-test for COVID-19 was high during the pandemic,^3^ COVID-19 fatigue and inertia may lessen motivation to test. We conducted a national survey to examine current intent to test for COVID-19.

## Methods

We conducted a cross-sectional online national survey between October 31 and November 7, 2024, using the Ipsos KnowledgePanel, which uses probability-based sampling to provide a nationally representative sampling frame for US adults. We asked about intent to conduct a home test if COVID-19 was suspected; respondents answering “no” or “not sure” were asked for reasons for not testing. Additional items assessed related attitudes and experiences (eAppendix in Supplement 1). This study followed the AAPOR reporting guideline.

All analyses used sample weights based on sex, age, race and ethnicity, census region, education, and household income. Ipsos collected demographic data, including race and ethnicity, via self-report to ensure sample representativeness. We used χ^2^ statistics to examine bivariate associations between intent to test and respondent characteristics and attitudes. We used forward stepwise selection to create a multivariate logistic regression model, starting with variables with *P* < .20 in bivariate analyses and retaining variables with *P* < .10. Analyses were conducted from November 14, 2024, to April 16, 2025, using SAS, version 9.4 (SAS Institute Inc). The UMass Chan Medical School institutional review board reviewed and approved this study and granted a waiver of written documentation of informed consent because the study activities involved no more than minimal risk and met the criteria for documentation not required outside of the research context. The Wald χ^2^ test was used to calculate 2-sided *P* values, with *P* = .05 the level used to indicate significance.

## Results

Of 2009 respondents to the question on COVID-19 self-testing, 1029 (51.2%) were female, and 244 (12.1%) were non-Hispanic Black; 361 (18.0%) were Hispanic; 185 (9.2%) were other, non-Hispanic or 2 or more races, non-Hispanic; and 1219 (60.7%) were non-Hispanic White, with mean (SD) age of 51.5 (18.4) years (Table 1). The survey completion rate was 63.4% (2016 of 3182).

**Table 1.** Respondent Characteristics and Bivariate Associations With Intent to Test If COVID-19 Is Suspected^a^

Characteristic	No. (%)			Overall *P* value determined by χ^2^ test
	Total sample (N = 2009)	Yes would test (n = 1407)	No or not sure would test (n = 602)	
Age, y				
18-29	396 (19.7)	248 (62.6)	148 (37.4)	<.001
30-44	524 (26.1)	358 (68.3)	166 (31.7)	
45-59	466 (23.2)	323 (69.3)	143 (30.7)	
≥60	623 (31.0)	478 (76.8)	145 (23.2)	
Education				
No high school diploma or GED	186 (9.3)	116 (62.3)	70 (37.7)	.04
High school diploma or GED	576 (28.7)	399 (69.2)	177 (30.8)	
Some college or associate’s degree	526 (26.2)	367 (69.8)	159 (30.2)	
Bachelor’s degree or higher	721 (35.9)	525 (72.8)	196 (27.2)	
Race and ethnicity				
Black, non-Hispanic	244 (12.1)	187 (76.5)	57 (23.5)	<.001
Hispanic	361 (18.0)	279 (77.4)	82 (22.6)	
Other, non-Hispanic or ≥2 races, non-Hispanic	185 (9.2)	144 (77.6)	41 (22.5)	
White, non-Hispanic	1219 (60.7)	797 (65.4)	422 (34.6)	
Language of survey completion				
English	1904 (94.8)	1324 (69.5)	580 (30.5)	.04
Spanish	105 (5.2)	83 (79.1)	22 (20.9)	
Sex				
Male	980 (48.8)	661 (67.4)	319 (32.6)	.01
Female	1029 (51.2)	746 (72.5)	283 (27.5)	
Marital status				
Married	1030 (51.3)	720 (69.9)	310 (30.1)	.90
Not married (includes widowed, divorced, separated, and never married)	979 (48.7)	687 (70.2)	292 (29.8)	
Household size, No.				
1	330 (16.4)	235 (71.2)	95 (28.8)	.03
2	710 (35.3)	520 (73.2)	190 (26.8)	
≥3	969 (48.2)	652 (67.3)	317 (32.7)	
Household income, $				
<24 999	197 (9.8)	147 (74.5)	50 (25.5)	.33
25 000-49 999	287 (14.3)	211 (73.4)	76 (26.6)	
50 000-74 999	295 (14.6)	205 (69.6)	90 (30.5)	
75 000-99 999	251 (12.5)	179 (71.4)	72 (28.6)	
100 000-149 999	388 (19.3)	264 (68.0)	124 (32.0)	
≥150 000	591 (29.5)	401 (67.8)	190 (32.2)	
Metropolitan Statistical Area status				
Nonmetropolitan	265 (13.2)	160 (60.3)	105 (39.7)	<.001
Metropolitan	1744 (86.8)	1247 (71.5)	497 (28.5)	
Census region				
Northeast	345 (17.1)	242 (70.2)	103 (29.8)	.02
Midwest	411 (20.5)	263 (63.9)	148 (36.1)	
South	778 (38.7)	556 (71.5)	222 (28.5)	
West	475 (23.7)	346 (72.8)	129 (27.2)	
Employment status				
Working (full or part time)	1259 (62.6)	869 (69.0)	390 (31.0)	.21
Not working	750 (37.4)	538 (71.7)	212 (28.3)	
Have a primary care physician				
Yes	1705 (84.9)	1229 (72.1)	476 (27.9)	<.001
No	303 (15.1)	178 (58.6)	125 (41.4)	
Self-rated health				
Excellent	222 (11.1)	135 (61.0)	87 (39.0)	<.001
Very good	722 (36.2)	481 (66.6)	241 (33.4)	
Good	756 (37.9)	559 (74.0)	197 (26.0)	
Fair	254 (12.8)	193 (75.8)	61 (24.2)	
Poor	42 (2.1)	29 (69.4)	13 (30.6)	
Trust in health care				
Do not trust at all	140 (7.1)	51 (36.4)	89 (63.6)	<.001
Trust a little	534 (26.8)	321 (60.0)	213 (40.0)	
Trust somewhat	907 (45.6)	680 (74.9)	227 (25.1)	
Trust a great deal	410 (20.6)	344 (83.8)	66 (16.2)	
Depend on numbers and statistics to make decisions about health				
Strongly agree	296 (15.0)	234 (79.0)	62 (21.0)	<.001
Somewhat agree	880 (44.5)	644 (73.2)	236 (26.8)	
Somewhat disagree	474 (24.0)	317 (66.9)	157 (33.1)	
Strongly disagree	327 (16.5)	191 (58.5)	136 (41.5)	
Heard of COVID-19 medications, such as Paxlovid				
Yes	1394 (70.1)	1022 (73.3)	372 (26.7)	<.001
No	596 (30.0)	375 (62.8)	221 (37.2)	
Have done a home test for COVID-19				
Yes	1416 (70.9)	1157 (81.7)	259 (18.3)	<.001
No	582 (29.1)	242 (41.6)	340 (58.4)	

^a^ All numbers of respondents were calculated using sample weights and rounded to the nearest integer. Percentages were calculated based on the exact weighted (noninteger) number. The survey margin of sampling error (MOSE) was 2.29% at the 95% confidence level for a point estimate equal to 50% (when the MOSE is at its widest) for the full sample.

Most respondents (70.0% [1407 of 2009]) would test if they suspected COVID-19. Several variables were associated with intent to test in bivariate analyses (Table 1). On multivariate analysis (Table 2), age older than 60 years; identifying as non-Hispanic Black, Hispanic, or other or multiple races, non-Hispanic; reporting other than excellent health (good, fair, or poor); having higher trust in the health care system; strongly agreeing they depend on numbers to make decisions about health; and having previously completed a COVID-19 home test were all associated with being more likely to test.

**Table 2.** Multivariate Logistic Regression Model of Factors Associated With Intent to Test If Suspected COVID-19

Characteristic	Odds ratio (95% CI)	*P* value
Age, y		
18-29	1 [Reference]	NA
30-44	1.37 (0.97-1.92)	.07
45-59	1.32 (0.93-1.86)	.12
≥60	2.14 (1.51-3.01)	<.001
Education		
No high school diploma or GED	1 [Reference]	NA
High school diploma or GED	1.63 (1.06-2.51)	.03
Some college or associate’s degree	1.23 (0.80-1.91)	.34
Bachelor’s degree or higher	1.23 (0.80-1.90)	.35
Race and ethnicity		
White, non-Hispanic	1 [Reference]	NA
Black, non-Hispanic	2.64 (1.79-3.90)	<.001
Hispanic	2.21 (1.58-3.10)	<.001
Other, non-Hispanic or ≥2 races, non-Hispanic	2.21 (1.41-3.46)	<.001
Census region		
Northeast	1 [Reference]	NA
Midwest	0.93 (0.65-1.35)	.72
South	1.39 (0.99-1.95)	.06
West	1.23 (0.85-1.79)	.28
Self-rated health		
Excellent	1 [Reference]	NA
Very good	1.01 (0.69-1.48)	.97
Good	1.74 (1.18-2.56)	.01
Fair	2.23 (1.37-3.62)	.001
Poor	2.54 (1.11-5.83)	.03
Trust in health care		
Do not trust at all	1 [Reference]	NA
Trust a little	1.64 (1.03-2.59)	.04
Trust somewhat	3.14 (2.00-4.90)	<.001
Trust a great deal	5.65 (3.39-9.41)	<.001
Depend on numbers and statistics to make decisions about health		
Strongly agree	1 [Reference]	NA
Somewhat agree	0.84 (0.58-1.21)	.36
Somewhat disagree	0.62 (0.42-0.93)	.02
Strongly disagree	0.46 (0.30-0.71)	<.001
Have done a home test for COVID-19		
Yes	1 [Reference]	NA
No	0.16 (0.12-0.20)	<.001

Abbreviations: GED, General Educational Development; NA, not applicable.

The percentage of respondents endorsing each reason for not or possibly not testing were not seeing a reason to test (53.6%), believing it would not be helpful to know if COVID-19 positive (30.1%), not trusting test results (20.7%), anticipating it would not or might not occur to one to test (19.4%), preferring not to know (9.1%), not knowing where to get a test (5.8%), being unable to afford a test (4.9%), and “other” (8.3%).

## Discussion

Nearly one-third of US adults would not or might not test for suspected COVID-19, largely because they do not see value in testing. Test hesitancy may delay oral antiviral initiation and could result in missed opportunities to limit transmission. Efforts are needed to increase awareness of the value of testing. Limitations of this study include that respondents’ reported intent to self-test may vary from their actual behavior and we may not have captured the full set of reasons why respondents may not self-test.

## Notes

Supplement 1. **eAppendix.** Survey Items and Response Options

Supplement 2.
Data Sharing Statement
