# Supplementary material for: Intent to Test for COVID-19 in the Postpandemic Era
Source: JAMA Netw Open. 2025 Jun 30;8(6):e2518250. doi: 10.1001/jamanetworkopen.2025.18250 (PMC12210076; doi:10.1001/jamanetworkopen.2025.18250)
Supplement: Supplement 1. — eAppendix. Survey Items and Response Options [file jamanetwopen-e2518250-s001.pdf]

Supplemental Online Content

Fisher KA, Mazor KM, Antonelli MT, Pretz C, Zhou Y, Soni A. Intent to test for COVID-19 in the postpandemic era. *JAMA Netw Open*. 2025;8(6):e2518250. doi:10.1001/jamanetworkopen.2025.18250

eAppendix. Survey Items and Response Options

This supplemental material has been provided by the authors to give readers additional information about their work.

© 2025 Fisher KA et al. *JAMA Network Open.*

eAppendix. Survey Items and Response Options

The full survey instrument is available from the authors upon request.

Do you have a regular doctor or primary care provider?

1. Yes
2. No

Now that COVID has become more of a "regular" disease, we're interested in your views on testing and treatment for COVID.

If you thought you might have COVID, would you do a home test?

1. Yes
2. No
3. Not sure

IF YES

Where would you get a test?

1. I have tests already at home
2. I would go to the store to buy a test
3. I would order a test online
4. Some other place, please specify [**TEXT BOX]**

IF NO

What are the reasons you wouldn't test yourself for COVID?

1. I can't afford to buy a COVID test
2. I don't know where to get a COVID test
3. I don't trust the test results
4. It wouldn't occur to me to test myself
5. I don't see any reason to test myself
6. I would rather not know if I have COVID
7. I don't think it would be helpful to know if I was COVID positive
8. Other, please specify [**TEXT BOX]**

IF NOT SURE

What are the reasons you might not test yourself for COVID?

1. I am not sure I can afford to buy a COVID test
2. I am unsure where to get a COVID test
3. I don't trust the test results
4. It might not occur to me to test myself
5. I might not see any reason to test myself
6. I might rather not know if I have COVID
7. I might not find it helpful to know if I was COVID positive
8. Other, please specify [**TEXT BOX]**

© 2025 Fisher KA et al. *JAMA Network Open.*

Have you ever heard of medications for COVID, such as Paxlovid, a prescription medicine (a pill) for treating COVID?

1. Yes
2. No

Have you ever used a home test kit to test yourself for COVID?

1. Yes
2. No

How much do you agree or disagree about the following statement?
In general, I depend on numbers and statistics to help me make decisions about my health.

1. Strongly agree
2. Somewhat agree
3. Somewhat disagree
4. Strongly disagree

In general, would you say your health is…

1. Excellent
2. Very good
3. Good
4. Fair
5. Poor

How much do you trust the healthcare system to do what's right for you?

1. Do not trust at all
2. Trust a little
3. Trust somewhat
4. Trust a great deal

Race and Ethnicity data are collected via Ipsos (the survey vendor) upon enrollment:

Are you of Hispanic, Latino, or Spanish origin?

Select all answers that apply.

1. No, I am not
2. Yes, Mexican, Mexican-American, Chicano
3. Yes, Puerto Rican
4. Yes, Cuban, Cuban American
5. Yes, other Spanish, Hispanic, or Latino group (Please specify, for example Argentinean, Colombian, Dominican, Nicaraguan, Salvadoran, Spaniard, and so on)

© 2025 Fisher KA et al. *JAMA Network Open.*

What race or races do you consider yourself to be?

We appreciate your effort to describe your background using these U.S. Census Bureau categories even though they might not match perfectly.

Select all answers that apply.

1. White
2. Black or African American
3. American Indian or Alaska Native
4. Asian
5. Native Hawaiian or other Pacific Islander
6. A different race

Responses are then provided to researchers using the following categories:

Categories Provided	Coded
1 = White, non-Hispanic	*What race or races do you consider yourself to be?*<br>=White
2 = Black, non-Hispanic	*What race or races do you consider yourself to be?*<br>= Black or African American
3 = Other, non-Hispanic	*What race or races do you consider yourself to be?*<br>=American Indian or Alaska Native; Asian; Native Hawaiian or other Pacific Islander
4 = Hispanic*****	*Are you of Hispanic, Latino, or Spanish origin?*<br>=Mexican, Mexican-American, Chicano and/or<br>=Puerto Rican and/or<br>=Cuban, Cuban American and/or<br>=Other Spanish, Hispanic, or Latino group
5 = 2+ races, non-Hispanic	Multiple responses selected in response to *What race or races do you consider yourself to be?*

This takes precedence.

Ipsos manually codes open-ended responses to *What race or races do you consider yourself to be?* =A different race. For instance, if someone selects "White" and specifies "Italian" in the open-ended response, they are classified as White. However, if they specify "mixed" in the open- ended response, they are coded as 2+ races. The final classification depends on the text entered in the open-end.

© 2025 Fisher KA et al. *JAMA Network Open.*
