# Supplementary material for: Intent to Test for COVID-19 in the Postpandemic Era
Source: JAMA Netw Open. 2025 Jun 30;8(6):e2518250. doi: 10.1001/jamanetworkopen.2025.18250 (PMC12210076; doi:10.1001/jamanetworkopen.2025.18250)
Supplement: Supplement 2. — Data Sharing Statement [file jamanetwopen-e2518250-s002.pdf]

Data Sharing Statement

Fisher. Intent to Test for COVID-19 in the Postpandemic Era. *JAMA Netw Open*. Published June 30, 2025. doi:10.1001/jamanetworkopen.2025.18250

Data
Data available: No
